# Supplementary material for: Scanned optogenetic control of mammalian somatosensory input to map input-specific behavioral outputs
Source: eLife. 2021 Jul 29;10:e62026. doi: 10.7554/eLife.62026 (PMC8428846; doi:10.7554/eLife.62026)
Supplement: Supplementary file 1. — List of parts used in system. A Solidworks assembly, the optical system control and acquisition software, and behavioral analysis toolkit are available at https://github.com/browne-lab/throwinglight (Schorscher-Petcu and Browne, 2020). [file elife-62026-supp1.docx]

Figure 1 - table 1. List of components for the assembly of the optical system

| **Description** | **Part Reference Number** | **Notes** | **Number** |
| --- | --- | --- | --- |
|  |  |  |  |
| **Stimulation** |  |  |  |
| Laser, 473 nm 100 mW | 06-01 MLD, Cobolt | Mounted on heatsink | 1 |
| Laser heatsink | Custom |  | 1 |
| Alignment mirrors | BB1-E02, Thorlabs | *M1* and *M2* in Fig. 1 | 2 |
| Alignment mirror mounts | POLARIS-K1-H, Thorlabs | Mounts BB1-E02 mirrors (*M1* & *M2*) | 2 |
| Lens, 30 mm focal length | AC254-030-A, Thorlabs | *L1* in Fig. 1 | 1 |
| Zoom housing | SM1NR1, Thorlabs | Housing for AC254-030-A (*L1*) | 1 |
| Filter wheel | SCFW6, Thorlabs | Houses ND filters (six settings), *F* in Fig. 1 | 1 |
| ND filter, OD 0.5 | NE505B, Thorlabs | Housed in SCFW6 for 32% transmittance (setting 2) or with one NE510B for 3.2% transmittance (setting 5) |  |
| ND filter, OD 1.0 | NE510B, Thorlabs | Housed in SCFW6 for 10% transmittance (setting 3) or with one NE505B for 3.2% transmittance (setting 5) | 2 |
| ND filter, OD 1.3 | NE513B, Thorlabs | Housed in SCFW6 for 5% transmittance (setting 4) | 1 |
| ND filter, OD 2.0 | NE520B, Thorlabs | Housed in SCFW6 for 1% transmittance (setting 6) | 1 |
| Lens, 150 mm focal length | AC254-150-A-ML, Thorlabs | *L2* in Fig. 1 | 1 |
| Lens, 500 mm focal length | AC254-500-A-ML | *L3* in Fig. 1 | 1 |
| Lens mount | LMR1/M, Thorlabs | Mounts C254-150-A-ML (*L2*) and AC254-500-A-ML (*L3*) | 1 |
| Dichroic Mirror | FF776-Di01-25x36, Semrock | *DM* in Fig. 1 | 1 |
| Dichroic mirror mount | CM1-DCH/M, Thorlabs | Houses FF776-Di01-25x36 (*DM*) | 1 |
| End cap | SM1CP2, Thorlabs | Blocks one port of CM1-DCH/M | 1 |
| Mirror galvanometer system | GVSM002/M, Thorlabs | x-axis and y-axis mirror galvanometers (*GM*), mount, servos and power supply | 1 |
| Galvanometer mount | GCM002/M, Thorlabs | Houses GVSM002/M mirrors (*GM*) | 1 |
| Translation stage | DT12XYZ/M, Thorlabs | Mounts GCM002/M | 1 |
| Glass platform | Custom cut borosilicate, 230 x 230 x 10 mm | Mice are studied directly on glass platform | 1 |
| Mouse chambers | Custom, laser-cut acrylic |  |  |
|  |  |  |  |
| **Acquisition** |  |  |  |
| LEDs for FTIR | 12v 850 nm LEDs SMD3528-300-IR, 4.8W/m | Surrounds glass platform within aluminium frame | 1 |
| Camera, local high-speed NIR | acA2000-165umNIR, Basler | Acquires at 160 x 160 pixels, 1000 fps | 1 |
| Camera lens | LM12JCM, Kowa | *CL2* in Fig. 1. Attached to acA2000-165umNIR | 1 |
| Camera, widefield | acA1920-40um, Basler | Acquires at 160 x 160 pixels, 40 fps | 1 |
| Camera lens | LM6HC, Kowa | *CL1* in Fig. 1. Attached to acA1920-40um | 1 |
| LED power supply | 12V DC power | Powers LEDs for FTIR | 1 |
|  |  |  |  |
| **Mounting components** |  |  |  |
| Aluminium breadboard base | MB3045/M, Thorlabs |  | 1 |
| Post, 38 mm | RS1.5P/M, Thorlabs | Mounts DT12XYZ/M | 1 |
| Post holders, 30 mm | PH30/M, Thorlabs | Holders for TR30/M | 3 |
| Post holders, 30 mm | PH30E/M, Thorlabs | Holders for TR30/M | 4 |
| Posts, 30 mm | TR30/M, Thorlabs | Mounts POLARIS-K1-H (*M1*), CP02/M, SCFW6 (*F*), LMR1/M (*L2* & *L3*), CM1-DCH/M (*DM*) and camera mounts | 7 |
| Post holder, 20 mm | PH20/M, Thorlabs | Holders for TR20/M, mounted on RC1 | 1 |
| Post, 20 mm | TR20/M, Thorlabs | Mounts POLARIS-K1-H (*M2*) | 1 |
| Clamping forks | CF125C/M, Thorlabs |  | 3 |
| Zoom housing mount | CP02/M, Thorlabs | Mount for SM1NR1 | 1 |
| Rail | RLA075/M, Thorlabs | Mounts RC1 | 1 |
| Rail carrier | RC1, Thorlabs | Mounts alignment mirror (*M2*) on 20 mm post | 1 |
| Mounting bases | BA2/M, Thorlabs | Mounts acA2000-165umNIR and CM1-DCH/M | 2 |
| Camera mounts | Custom | Mounts acA2000-165umNIR and acA1920-40um | 2 |
| Cage cube connector | CM1-CC, Thorlabs | Connects GCM002/M to CM1-DCH/M | 1 |
|  |  |  |  |
| **Control** |  |  |  |
| Master computer | Custom | Controls system and acquires data | 1 |
| Multifunction I/O device | USB-6211, National Instruments | Interface between acquisition computer, galvanometers, Arduino UNO and laser | 1 |
| Arduino | Arduino UNO | Triggers USB-6211 if required | 1 |
| Analysis computer | Custom | Runs scripts and algorithms for quantitative behavioural analysis | 1 |
|  |  |  |  |
| **Frame components** |  |  |  |
| Frame rails | 200 x 25 x 25 mm aluminium extrusion |  | 4 |
| Frame rails | 375 x 25 x 25 mm aluminium extrusion |  | 2 |
| Frame rails | 225 x 25 x 25 mm aluminium extrusion |  | 4 |
| Frame rails | 275 x 25 x 25 mm aluminium extrusion |  | 6 |
|  |  |  |  |
| **Characterization** |  |  |  |
| Power meter | PM121D and S121C, Thorlabs | Measure light intensity at output from mirror galvanometers and at glass platform | 1 |
| Beam profiler | BP209-VIS/M, Thorlabs | Measure spot size at stimulation plane using alignment plates | 1 |
| Alignment plates | Custom aluminium plate, 275 x 275 x 13.9 mm | Allows accurate placement of BP209-VIS/M at multiple locations at stimulation plane | 1 |
| Grid array | R1L3S3P, Thorlabs | Calibration of galvanometer jump distance at stimulation plane | 1 |
